# Regulatory enforcement of the marketing of fixed-dose combinations in India: a case study of systemic antibiotics

**DOI:** 10.1186/s40545-023-00644-y

**Published:** 2023-11-10

**Authors:** Petra Brhlikova, Aashna Mehta, Patricia McGettigan, Allyson M. Pollock, Peter Roderick, Habib Hasan Farooqui

**Affiliations:** 1 Population Health Sciences Institute, Newcastle University, Baddiley-Clark Building, Newcastle Upon Tyne, NE2 4AX UK; 2 Public Health Foundation of India, Gurugram, India; 3 William Harvey Research Institute, Queen Mary University of London, London, UK; 4 College of Medicine, Qatar University, Doha, Qatar

**Keywords:** India, Regulation, Fixed-dose combination (FDC), Systemic antibiotics

## Abstract

**Background:**

In India, states have licensed the manufacture of large numbers of fixed-dose combination (FDC) drugs without the required prior approval of the central regulator. This paper describes two major regulatory initiatives to address the problem, which began in 2007 and 2013, and examines whether they have been sufficient to remove centrally unapproved systemic antibiotic FDCs from the market.

**Methods:**

Information was extracted from documents published by the central regulator and the ministry of health, including the National List of Essential Medicines (NLEM), and court judgments, and analysed alongside sales volume data for 2008–2020 using PharmaTrac market dataset.

**Results:**

The regulatory initiatives permitted 68 formulations to be given de facto approvals (‘No Objection Certificates’) outside the statutory regime, banned 46 FDCs and restricted one FDC. Market data show that FDCs as a proportion of total antibiotic sales increased from 32.9 in 2008 to 37.3% in 2020. The total number of antibiotic FDC formulations on the market fell from 574 (2008) to 395 (2020). Formulations with a record of prior central approval increased from 86 (2008) to 94 (2020) and their share of the antibiotic FDC sales increased from 32.0 to 55.3%. In 2020, an additional 23 formulations had been permitted de facto approval, accounting for 10.6% of the antibiotic FDC sales. Even in 2020, most marketed formulations (70.4%, 278/395) were unapproved or banned, and comprised a 15.9% share of the antibiotic FDC sales. The share of NLEM-listed antibiotic FDC sales increased from 21.2 (2008) to 26.7% (2020).

**Conclusion:**

The initiatives had limited impact. Regulatory enforcement has been slow and weak, with many unapproved, and even banned, FDCs remaining on the market.

**Supplementary Information:**

The online version contains supplementary material available at 10.1186/s40545-023-00644-y.

## Background

Regulation of medicines in India continues to be based on a division of responsibilities between the central government and the individual states (and Union territories), which is founded on legislation dating from 1940, and on its 1949 constitution.

Despite many amendments to the legislation, none have changed the fundamental distribution of functions. States grant licences for manufacturing, selling and distributing drugs, whilst licences for importing are granted centrally, now by the national regulator, the Central Drugs Standard Control Organisation (CDSCO) headed by the Drugs Controller General (India) (DCG(I)). This shared responsibility is also reflected in the constitution, in which ‘Drugs’ became and still is an item included in the ‘Concurrent List’, so giving both the national parliament and state legislatures power to make laws in relation to them. Only states can legislate for ‘Public health’ matters. Incrementally over time, the centre’s powers have increased. Since 1961, the rules have required the manufacture of ‘new drugs’, a concept introduced in 1952, to have been approved first by the central regulator [1]. From 2001, the rules required the central regulator to give approval when it was satisfied that the new drug was safe and effective [2], but new rules removed this requirement from March 2019 [3]. McGettigan et al. 2015 gives a fuller account of the regulatory system to 2015 [4].

Perhaps inevitably, this division of responsibility has resulted in much friction between the states and the centre, and has led to calls for the centre to have sole constitutional responsibility for drug regulation, and to share responsibility for public health with the states [5].

One example of where the division has caused difficulties, and public health concerns, has been in the regulation of fixed-dose combination drugs (FDCs). Official calls for them to be more stringently controlled date back over 40 years [6, 7]. Over many years, FDC manufacturing licences have been issued by states without prior CDSCO approval^4^ even where they fell within the definition of a ‘new drug’. Measures have been taken to address this problem, both by the Ministry of Health and Family Welfare (MoHFW) issuing statutory prohibition and restriction orders in 2016–19 [8–11], and by CDSCO permitting applications for issuing ‘No Objection Certificates’ (NOCs) outside the statutory regime [12, 13]. These followed recommendations by three technical committees [14–19] and successful legal actions by the industry [4, 20, 21].

Previous studies documented that India has the highest number of antibiotic FDCs on the market worldwide, and with many of them potentially inappropriate, the risk of increasing antimicrobial resistance is a particular and global concern [22–24]. In India, of the 4.5 billion standard units of antibiotic FDCs sold in 2020, 41.5% were attributed to combinations listed as ‘not recommended’ by WHO [25].

Our aim was to discover whether the measures taken by the government and central regulator were sufficient to remove centrally unapproved systemic antibiotic FDCs from the market. This paper provides an account of those technical committees and measures and examines the annual sales of systemic antibiotic FDCs between 2008 and 2020 in light of those measures.

## Methods

### Data sources

The analysis is based on the study of official documents and market sales data.

#### Official documents

Minutes of the meetings of the Drugs Technical Advisory Board (DTAB) and the Drugs Consultative Committee, available on the CDSCO website, were viewed in order to find references to the regulatory initiatives. We consulted the ‘Public Notices’ sections of the CDSCO website in order to identify steps taken to progress the initiatives. From the CDSCO website we also identified reports of the technical committees and FDCs which had been formally approved, permitted ‘No Objection Certificates’ (NOC), and banned (including gazette notifications). A list of the 294 FDCs considered by the Gupta committee was obtained from CDSCO following a ‘Right to Information’ request. The National Lists of Essential Medicines were identified by internet searches. Court judgments were located by searching https://indiankanoon.org, a legal database. The analysed documents consisted of: (1) the lists published by CDSCO of all FDCs (i) approved since 1961 to 31st December 2019 [26], and in 2020 [27]^27^; (ii) permitted an NOC, published on 8th January 2020 [12] and on the 27th August 2021 [13]; (iii) prohibited for manufacture and sale through Gazette notifications under section 26A of the Drugs & Cosmetics Act 1940 from July 1983 to June 2017 [28], in September 2018 [10], and January 2019 [11]; (2) the National List of Essential Medicines (NLEM) 2011 [29] and 2015 [30]; (3) the reports of two technical committees [14–17, 19] and the summary of the report of one such committee [18]; (4) minutes of meetings of the statutory Drugs Technical Advisory Board and Drugs Consultative Committee; and (5) judgments of the Delhi High Court [20] and of the Supreme Court of India [21].

#### Market sales data for the period 2008–2020

‘PharmaTrac’ is a pharmaceutical sales dataset compiled by market research company All Indian Origin Chemists & Distributors Limited Pharmasofttech [31]. Data are collected from a sample of 10,000 stockists across 30 different regions of the country and then projected to reflect the overall sales in the private pharmaceutical market in India. The dataset captures drug names, dosage form and strength of marketed products, and their sales in terms of volume and value. The analyses in this paper are based on volumes only. Volumes reported in packs, were recalculated to Standard Units (SUs), where one SU was one tablet/capsule, one injection vial, or one bottle of oral medicine.

#### Identification and coding of FDCs

From official documents (1)–(3) and market data, we identified systemic antibiotic FDCs. Fixed-dose combination products comprise two or more drugs combined in a fixed ratio of doses and available in a single dosage form. We included FDCs comprising one antibiotic and non-antimicrobial agent(s), and combinations of an antibiotic with another antibiotic or with an antimicrobial agent (dual antimicrobials). We excluded: systemic antimicrobial FDCs that did not include any antibiotic, e.g., anti-virals, anti-fungals; FDCs indicated for tuberculosis where there is a well-established evidence base for FDC use; topical preparations, kits and combikits (packaging including two or more medicines to be used concomitantly). Data sources varied in whether they refer only to combinations, or to combinations with specific dosage forms and strengths (formulations). Formal CDSCO approvals, effective approvals via NOCs and NLEM listing refer to FDC formulations with specific dosage forms and strengths. Bans referred mostly to combinations, with three exceptions where dosage form and/or strength was specified. For details of our approach to coding see Additional file 1: Data coding.

Where the PharmaTrac dataset did not provide information on strength, or grouped products according to an antibiotic class or therapeutic group, CDSCO approval, NOC and NLEM listing could not be determined. We defined these as ‘Undetermined'.

### Analysis of sales data

We generated numbers, volume, and market share for: (1) systemic antibiotic FDCs and single drugs (SDs), and (2) for FDC formulations (i) with formal CDSCO approval, (ii) permitted an NOC, (iii) banned, (iv) unapproved, and (v) listed on the NLEM.

## Results

### Regulatory initiatives and measures

Two significant sets of measures have been taken by the government and CDSCO under separate initiatives which began in 2007 and 2013 (see Table 1).

**Table 1:** Milestones in initiatives begun in 2007 and 2013 to control proliferation of FDCs

	First initiative (2007)	Second initiative (2013)
2007, November	States directed by MoHFW to cancel licences for 294 centrally unapproved FDCs (section 33P). Directions stayed by Madras High Court	
2013, January		CDSCO sends “18 month policy decision” letters to state regulators
2014, January		Suresh Committee, and 10 Expert Committees set up
2014, September		Kokate Committee established, replacing Suresh Committee and 10 Expert Committees
2015, January		1st Kokate report published
2015, February	Summary of the evaluation of the 294 FDCs by the Gupta committee published	
2015, April		2nd Kokate report published
2015, July		CDSCO issues first NOCs
2016, February		3rd Kokate report published
2016, March		Government bans (section 26A). Immediately stayed temporarily by Delhi High Court
2016, May		4th Kokate report
2016, December		Government bans ruled unlawful by Delhi High Court
2017, June		Government bans (section 26A)
2017, December	Supreme Court accepts Gupta committee report	Supreme Court set aside the judgment of the Delhi High Court, but remitted the matter to the DTAB
2018, February		DTAB sets up Kshirsagar committee
2018, July		Kshirsagar committee report published
2018, September		Government bans (section 26A)
2019, January	Government bans (section 26A)	

*NOC*  No Objection Certificate, *DTAB*  Drugs Technical Advisory Board. References to sections are to sections of the Drugs and Cosmetics Act 1940, as amended

#### First initiative in 2007

In June 2007, the statutory Drugs Consultative Committee was briefed by the DCG(I) on a reference from the Prime Minister’s Office (PMO) concerning irrational and unapproved FDCs, which was described as a “perennial problem” requiring a solution [32].

In November 2007, the central government directed state drug regulators to cancel licences they had issued for the manufacture of 294 FDCs including 52 unique systemic antibiotic FDCs which had not had central approval [11]. This action followed complaints from Consumer Associations [33] and a DCG(I) examination [32], and a presumably unsuccessful prior request by the DCG(I) to states to withdraw the licences [32]. It is unknown how the list of 294 was drawn up.

Immediate legal challenges by the industry persuaded the Madras High Court to stay the effect of these directions, enabling the FDCs to remain on the market. A sub-committee of the statutory Drugs Technical Advisory Board (DTAB) was set up, apparently in 2008, to examine these FDCs ‘on a fast tract (*sic*) basis’ [34]. Seven years later, in February 2015, a summary of the assessment report of that sub-committee, chaired by Professor Y.K. Gupta, was published [18].

#### Second initiative in 2013

Following publication of a highly critical parliamentary report in May 2012 [35], a separate initiative was begun in January 2013. The DCG(I) wrote letters to state authorities asking them to request manufacturers within 18 months to prove the safety and efficacy of FDCs which fell within the definition of a ‘new drug’ and which the state authorities had licensed before 1st October 2012 without permission of the central regulator [36]. Prosecutions of companies which continued to manufacture such FDCs were not systematically instituted and they remained on the market.

By November 2013, over 5000 ‘applications’ had been submitted by manufacturers [37]. Ten Expert Committees were set up to consider the submitted FDCs [38]. The DTAB set up a sub-committee chaired by Dr. B. Suresh, President of the Pharmacy Council of India to prepare guidelines for the Expert Committees to follow [39].

This route, however, was subsequently aborted following concerns expressed by manufacturers. In July 2014, the President of the Indian Drugs Manufacturers’ Association wrote to the MoHFW stating that “India is the world leader in FDCs” and suggesting that the Expert Committees were “strongly biased against advocating combinations” [40]. In September 2014, a committee chaired by Professor C.K. Kokate was established by the Ministry, replacing the Suresh and Expert Committees [14].

Over 6000 FDC formulations were eventually submitted to the Kokate committee. It published four reports between January 2015 and May 2016 (Table 1). In July 2015, CDSCO began a process for issuing ‘No Objection Certificates’ (NOCs) for formulations of FDCs which the committee had assessed as ‘rational’. It appears that manufacturers were entitled to apply for NOCs which, when issued, would amount to a de facto or effective approval of the formulations outside the statutory regime and without a formal CDSCO approval. A total of 68 systemic antibiotic FDC formulations were allowed NOCs in 2015–2017 and 2020 (Additional file 1: Table A1). However, how many manufacturers have applied for NOCs remains unknown.

The third Kokate committee report in February 2016 set out its then final evaluation of formulations which it considered irrational, referring to the “serious concern in the country” of antibiotic resistance from “injudicious use”. In March 2016 the government prohibited the manufacture, sale and distribution of 344 FDCs and formulations including 35 systemic antibiotic (Additional file 1: Table A2). Immediate legal challenges again by the industry led to a temporary stay of the bans in the Delhi High Court. The stay was confirmed by the court in December 2016 on the basis that the DTAB should have been consulted. An appeal was made to the Supreme Court.

#### The initiatives converge in the Supreme Court in 2017

In December 2017, the Supreme Court held that it was not necessary for the DTAB to have been consulted and set aside the judgment of the Delhi High Court. However, the court ordered 334 FDCs and formulations to be examined by the DTAB. These consisted of the 344 banned in 2016, excluding 15 licensed before 21 September 1988, plus five which had been banned in 2017. The court set aside the banning orders for the 15 FDCs. The five banned in 2017 included an additional dosage form of an antibiotic banned in 2016 and one additional antibiotic FDC. The Supreme Court also accepted the assessment report on the FDCs that had been evaluated by the Gupta committee.

#### Since 2018

The examination ordered by the Supreme Court was conducted by a DTAB sub-committee chaired by Dr. Nilima Kshirsagar. This committee reported in July 2018, and the government issued fresh bans and restrictions for 334 FDCs and formulations in September 2018.

These included 33 of the 35 antibiotics prohibited in March 2016, plus a restriction on one formulation (amoxycillin + potassium clavulanate), which had been banned in 2016 and 2017 (Additional file 1: Table A2). Amoxycillin + bromhexine FDC was not banned in 2018 as its manufacture had been licensed before 21 September 1988, although the Kshirsagar committee recommended its prohibition.

In January 2019, a further 80 FDCs including 11 systemic antibiotics were banned, following the recommendations of the Gupta committee (Additional file 1: Table A2).

In April 2019, a fifth report from the Kokate committee, was put before the DTAB [41]. This report confirmed findings of irrationality for over 400 formulations which the committee had evaluated as irrational in its fourth report in May 2016. The DTAB set up another sub-committee, again chaired by Dr. Kshirsagar, to evaluate those formulations. Neither the fifth report of the Kokate committee, nor of the sub-committee, have been published. The process of issuing NOCs continued beyond the study period [42].

### Analyses of market sales

(1) Antibiotic FDCs and single drugs in India, 2008–2020

Total sales of antibiotics increased by volume between 2008 and 2015 and then declined (Table 2). However, FDC volumes in 2020 were 23.3% higher than in 2008, accounting for a larger proportion of the market in 2020 (37.3%) than in 2008 (32.9%), whilst single drug volumes grew by 1.5% over the same period. 114 FDCs were marketed in 2008 increasing to 123 in 2014 and decreasing to 112 in 2020.

**Table 2:** Number, sales volume (in billion Standard Units), and proportion (%) of FDCs and single drugs (SDs) on the Indian antibiotic market, 2008–2020

		2008	2009	2010	2011	2012	2013	2014	2015	2016	2017	2018	2019	2020
FDCs	No.	114	115	119	122	124	121	123	119	119	118	116	116	112
	Vol	3.7	4.0	4.2	4.5	4.8	5.3	5.5	5.9	5.6	5.3	5.0	4.9	4.5
		32.9%	33.6%	33.9%	35.2%	36.8%	37.8%	40.0%	41.6%	39.5%	39.2%	37.7%	36.9%	37.3%
SDs	No.	78	78	77	79	77	79	80	78	76	77	77	78	79
	Vol	7.5	7.8	8.3	8.2	8.3	8.8	8.3	8.3	8.5	8.2	8.3	8.4	7.6
		67.1%	66.4%%	66.1%%	64.8%	63.2%	62.3%%	60.0%%	58.4%	60.5%%	60.8%	62.3	63.19%	62.7%
Total	Vol	11.1	11.8	12.5	12.7	13.1	14.2	13.8	14.2	14.1	13.4	13.2	13.3	12.1

(2) Antibiotic FDCs

Between 2008 and 2020, there were 143 different antibiotic FDCs (Additional file 1: Table A3) on the market of which 79 (55.2%) were dual antimicrobials. The 143 FDCs were marketed in 817 different formulations, with the highest number of FDC formulations marketed in 2011 at 646 and the lowest number in 2020 at 395 (Table 3).

**Table 3:** Number, volume (in billion SUs), and market share of antibiotic FDC formulations marketed in India by approval and ban status, 2008–2020

		2008	2009	2010	2011	2012	2013	2014	2015	2016	2017	2018	2019	2020
FDCs	No.	114	115	119	122	124	121	123	119	119	118	116	116	112
	Vol	3.7	4.0	4.2	4.5	4.8	5.3	5.5	5.9	5.6	5.3	5.0	4.9	4.5
FDC formulations	No.	574	598	634	646	611	599	578	531	526	508	486	421	395
Formal CDSCO Approval	No.	86	93	100	107	108	113	111	109	106	106	103	96	94
		15.0%	15.6%	15.8%	16.6%	17.7%	18.9%	19.2%	20.5%	20.2%	20.9%	21.2%	22.8%	23.8%
	Vol	1.2	1.4	1.7	2.0	2.3	2.4	2.5	2.6	2.7	2.7	2.7	2.7	2.5
		32.0%	35.2%	40.9%	45.1%	47.5%	45.1%	46.1%	44.7%	48.8%	52.2%	53.3%	54.1%	55.3%
Effective Approval (NOC)	No.	–	–	–	–	–	–	–	16	20	22	20	20	23
		–	–	–	–	–	–	–	3.0%	3.8%	4.3%	4.1%	4.8%	5.8%
	Vol	–	–	–	–	–	–	–	0.5	0.6	0.5	0.5	0.5	0.5
		–	–	–	–	–	–	–	8.0%	10.1%	9.8%	9.4%	9.5%	10.6%
Approved TOTAL	No.	86	93	100	107	108	113	111	125	126	128	123	116	117
		15.0%	15.6%	15.8%	16.6%	17.7%	18.9%	19.2%	23.5%	24.0%	25.2%	25.3%	27.6%	29.6%
	Vol	1.2	1.4	1.7	2.0	2.3	2.4	2.5	3.1	3.3	3.3	3.1	3.1	3.0
		32.0%	35.2%	40.9%	45.1%	47.5%	45.1%	46.1%	52.7%	58.9%	61.9%	62.7%	63.7%	65.9%
Banned	No.	0	0	0	0	0	–	–	–	60	3	47	45	39
		< 0.1%	< 0.1%	< 0.1%	< 0.1%	< 0.1%	–	–	–	11.4%	0.6%	9.7%	10.7%	9.9%
	Vol	< 0.1	< 0.1	< 0.1	< 0.1	< 0.1	–	–	–	0.3	< 0.1	0.2	< 0.1	< 0.1
		< 0.1%	< 0.1%	< 0.1%	< 0.1%	< 0.1%	–	–	–	4.9%	0.1%	3.9%	0.7%	0.2%
Unapproved	No.	488	505	534	539	503	486	467	406	340	377	316	260	239
		85.0%	84.4%	84.2%	83.4%	82.3%	81.1%	80.8%	76.5%	64.6%	74.2%	65.0%	61.8%	60.5%
	Vol	2.3	2.4	2.4	2.3	2.4	2.3	2.3	1.8	1.3	1.3	1.0	0.8	0.7
		63.3%	61.6%	56.0%	51.5%	49.8%	43.9%	41.3%	31.4%	22.7%	25.5%	19.5%	16.6%	15.7%
Banned and unapproved TOTAL	No.	489	506	535	540	504	487	467	406	400	380	363	305	278
		85.2%	84.6%	84.4%	83.6%	82.5%	81.3%	80.8%	76.5%	76.0%	74.8%	74.7%	72.4%	70.4%
	Vol	2.3	2.4	2.4	2.3	2.4	2.3	2.3	1.8	1.5	1.3	1.2	0.9	0.7
		63.3%	61.7%	56.1%	51.5%	49.9%	43.9%	41.3%	31.4%	27.6%	25.6%	23.4%	17.4%	15.9%
Undetermined	Vol	0.2	0.1	0.1	0.2	0.1	0.6	0.7	0.9	0.8	0.7	0.7	0.9	0.8
		4.7%	3.2%	3.1%	3.4%	2.6%	11.0%	12.6%	15.9%	13.6%	12.5%	13.9%	19.0%	18.2%

‘Undetermined’ include sales of FDCs for which the PharmaTrac dataset did not provide information on strength, or grouped products according to an antibiotic class or therapeutic group. Due to this data limitation, CDSCO approval and NOC could not be determined for 3–19% of market sales by volume in the study period

(i) FDC formulations with formal CDSCO approval

Among the 817 FDC formulations marketed during 2008–2020, 129 (15.8%) had a record of prior formal approval. The number of formally approved formulations on the market decreased between 2013 and 2020 while the total number of FDC formulations decreased more markedly (Table 3). As a result, the proportion of formally approved formulations increased from 18.9% (113/599) in 2013 to 23.8% (94/395) in 2020. Overall, the market share of formally approved formulations increased from 32.0% to 55.3% between 2008 and 2020, respectively.

(ii) FDC formulations permitted NOCs

In 2015, 16 FDC formulations on the market had NOCs rising to 23 in 2020. Their market share by volume increased from 8.0% to 10.6% in the same period (Table 3). There were 28 formulations with NOCs for which no sales were recorded (Additional file 1: Table A1). Effective approval of FDC formulations using NOCs, brought the overall proportion of approved FDC formulations on the market to 29.6% (117/395) in 2020 from 15.0% (86/574) in 2008 (Table 3). All approved formulations accounted for 32.0% and 65.9% of sales volume in 2008 and 2020, respectively (Table 3).

(iii) Banned FDCs

In 2008–2012 there were traces on the market of FDCs banned before 2008, although their market volume was small (Table 3). 48 different systemic antibiotic FDC were banned and restricted in use in 2016–2019, with more than half being dual antimicrobials (Additional file 1: Table A2).

*First initiative in 2007*

The first initiative to control proliferation of FDCs resulted in 11 FDCs being banned over 11 years later in January 2019 (Additional file 1: Table A2). Six of the 11 FDCs banned in 2019 were not marketed in the study period. Five banned combinations were marketed in nine formulations in 2019, with four combinations in eight formulations remaining on the market in 2020 (Table 4). Their market share by volume, although very small, increased until 2018 and declined only after the 2019 ban.

**Table 4:** Number, sales volume (in million SUs) and proportion (%) of the systemic antibiotic FDC formulations banned as a result of the first initiative

Formulations		2008	2009	2010	2011	2012	2013	2014	2015	2016	2017	2018	2019	2020
Banned in 2019	No	8	8	9	10	10	10	10	10	9	9	9	9	8
		1.4%	1.3%	1.4%	1.5%	1.6%	1.7%	1.7%	1.9%	1.7%	1.8%	1.9%	2.1%	2.0%
	Vol	11.2	14.4	19.3	24.6	31.0	37.8	45.3	49.3	46.1	47.6	47.6	11.2	3.7
		0.3%	0.4%	0.5%	0.6%	0.6%	0.7%	0.8%	0.8%	0.8%	0.9%	1.0%	0.2%	0.1%

Volumes are expressed in millions because the number of banned formulations was low

*Second initiative in 2013*

The second initiative resulted in 35 FDCs being banned in 2016, and an additional two FDCs being banned in 2017. In 2018, 34 of these 36 FDCs were banned again, the use of one was restricted, and one was neither banned again nor restricted (Additional file 1: Table A2). 60 marketed formulations were banned in 2016 accounting for about 5% of the systemic antibiotic FDC market by volume (Table 5). That share had begun to decline two years before the ban, and continued to decline in 2017 despite the ban having been immediately stayed. The 2017 ban accounted for less than 1% of the total systemic antibiotic FDC market by volume (three marketed formulations). About 4% of the market volume was banned in 2018 (47 marketed formulations), falling to 0.1% by 2020.

**Table 5:** Number, sales volume (in million SUs) and proportion (%) of the systemic antibiotic FDC formulations banned as a result of the second initiative

Formulations		2013	2014	2015	2016	2017	2018	2019	2020
Banned in 2016	No.	68	68	59	60	57	52	37	
		11.4%	11.8%	11.1%	11.4%	11.2%	10.7%	8.8%	
	Vol	345.2	365.1	382.3	271.9	229.6	195.8	25.6	
		6.5%	6.6%	6.5%	4.9%	4.4%	3.9%	0.5%	
Banned in 2017	No.		2	2	3	3	3	3	2
			0.3%	0.4%	0.6%	0.6%	0.6%	0.7%	0.5%
	Vol		5.4	5.6	5.8	3.9	3.3	0.8	< 0.1
			0.1%	0.1%	0.1%	0.1%	0.1%	< 0.1%	< 0.1%
Banned in 2018	No.			52	53	51	47	36	31
				9.8%	10.1%	10.0%	9.7%	8.6%	7.8%
	Vol			382.0	272.7	228.7	195.6	23.8	5.9
				6.5%	4.9%	4.4%	3.9%	0.5%	0.1%

Volumes are expressed in millions because the number of banned formulations was low

For 17/34 (50.0%) combinations banned in 2018 no sales were recorded during the study period (Additional file 1: Table A2). However, 16 of the 17 FDCs with market data remained on the market in at least 31 formulations in 2020 (Additional file 1: Table A4), although with a decreasing sales volume and market share (Table 5).

(iv) Unapproved (and not banned) FDC formulations

**Fig. 1:**
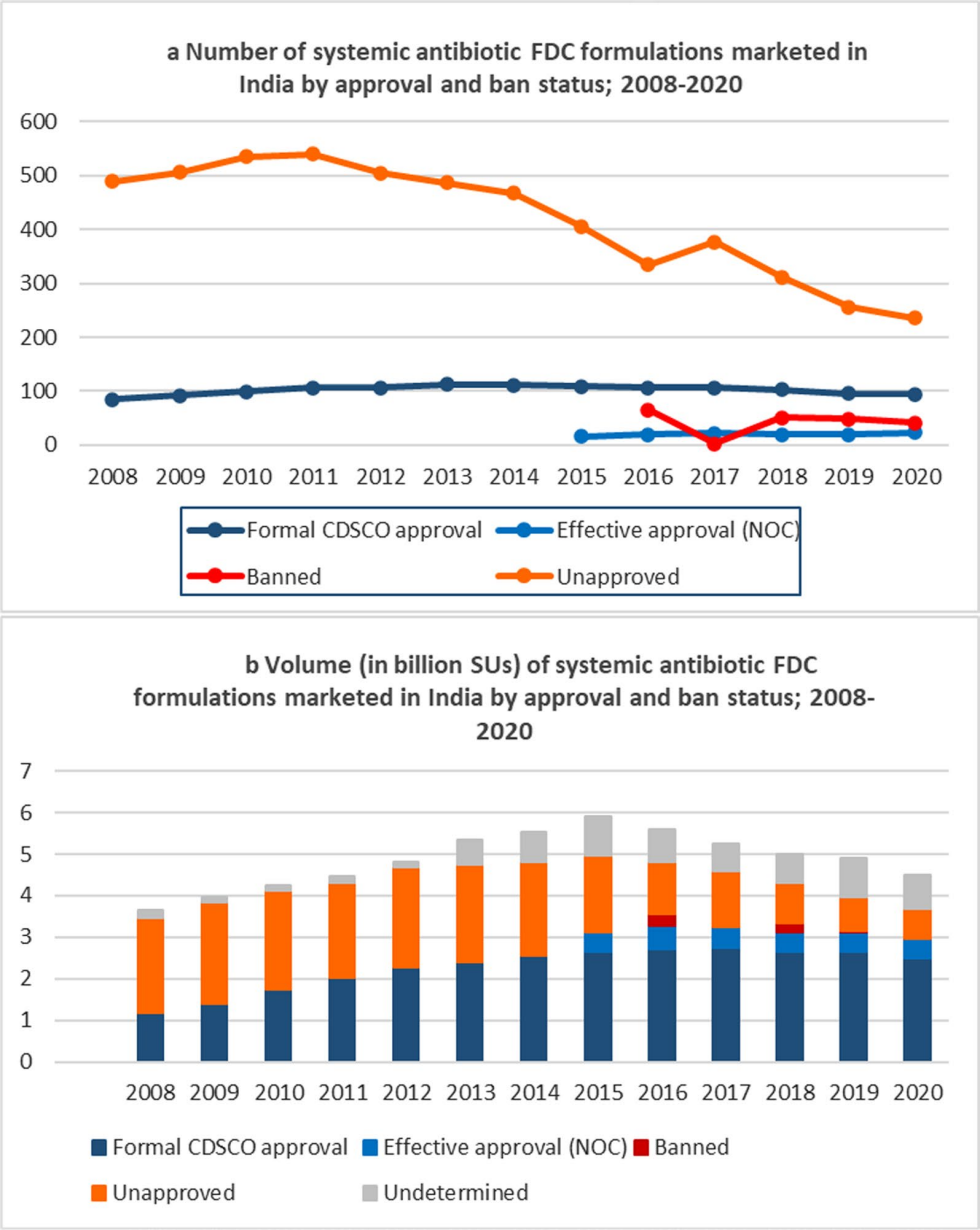
Systemic antibiotic FDC formulations marketed in India by approval and ban status, 2008–2020

From 2008 to 2020 the unapproved formulations decreased in number from 488 to 239 (Additional file 1: Table A5) and their market share by volume decreased from 63.3% to 15.7% (Table 3). Nevertheless, in every year of the study period the majority of marketed FDC formulations were unapproved (Fig. 1a).

(v) NLEM-listed FDC formulations

Of the 817 marketed FDC formulations, 20 (2.4%) were listed on NLEM2011, NLEM2015, or both. 13/20 (65.0%) had a record of formal CDSCO approval and NOCs were permitted for two additional formulations in 2015 (Additional file 1: Table A6). The number of NLEM-listed FDC formulations on the market increased from 2% (10/574) to 4% (14/395) between 2008 and 2020 (Additional file 1: Table A7). Their overall market share rose from 21.2% to 26.7% by volume during 2008–2020.

## Discussion

India has long-acknowledged that its large numbers of centrally unapproved FDCs and vast sales of many of these are matters of public health concern because they were not assessed for safety and efficacy by CDSCO. In the context of increasing antimicrobial resistance, antibiotic FDCs are of particular and global importance [22–25]. Our study establishes that despite regulatory initiatives and measures since 2007 to control sales, hundreds of unapproved antibiotic FDC formulations remained on the Indian market accounting for over 700 million of the 4.5 billion standard units sold in 2020. An additional one-third of standard units (1.5/4.5 billion) were of antibiotic FDCs approved in India but not recommended by the WHO [25].

Limited and delayed progress has been made in this therapeutic area. Formally approved systemic antibiotic FDCs accounted for about 55% of volume in 2020, compared with just under a third in 2008. An additional 11% of volume in 2020 was approved outside the statutory regime (NOCs). After rising to 646 in 2011, the number of formulations on the market fell to 395 in 2020. Sale volumes of FDCs banned in 2018 and 2019 had fallen substantially by 2020. It is encouraging that the limited set of FDCs prioritised for public procurement has a significant presence also on the private market: the share of NLEM-listed systemic antibiotic FDC formulations on the market increased from 2% in 2011 to 4% by number and from 21 to 27% by volume.

However, despite the number of formulations reducing, in 2020 some two thirds (239/395) of marketed formulations [Additional file 1: Table A5], accounting for over one-sixth (16%) by volume, were still neither formally approved nor de facto approved with an NOC. And 39 formulations banned in 2018 and 2019 remained on the market in 2020. Given recent research findings of high sales in 2019 of FDCs not recommended by WHO [43] and of 13 of the 20 top-selling systemic antibiotic FDCs in 2020 being expressly not recommended by the WHO [25], the higher overall market share of systemic antibiotic FDCs in 2020 (37%) compared to 2008 (33%), is a cause for serious public health concern.

This slow and limited progress is the result of weak, convoluted, badly targeted and inefficient regulatory enforcement. CDSCO undertook limited monitoring of the approval status of products in the market [32] before the government directed that manufacturing licences should be cancelled in 2007. The government did not continue to defend the litigation in the Madras High Court and the FDCs were referred to the Gupta committee for assessment, which took about seven years to report. There is no evidence that any market surveillance was conducted before the second initiative was begun in 2013, and its scope was restricted by giving the industry control over which FDCs would be assessed by a committee. The industry then lobbied successfully to prevent the Suresh and 10 expert committees continuing their work, and later successfully litigated to prevent the 2016 bans based on the recommendations of the Kokate committee from being implemented. The subsequent adoption by CDSCO of a de facto approval route for FDCs which the committees assessed as ‘rational’, then regularised the irregular. The meaning of rationality as used by the committees and CDSCO is unclear, as is its relationship to the express statutory requirements (until March 2019) [3] that CDSCO must, before giving its approval, be satisfied that the drug is safe and effective (*sic*). Removal of that requirement in 2019 is extremely worrying as the threshold standard for new drug approvals is now unspecified and unclear. Draft policy proposals which purport to elaborate the 2019 rules do not change the legal position, even if adopted [44]. There is no evidence that systematically prosecuting companies which continued to manufacture FDCs that required but did not have central approval was considered, as recommended by Sharma [5].

One result of the approach was that the committees assessed, the government banned and CDSCO permitted approvals outside the statutory regime for antibiotic formulations which were not marketed during the study period (Additional file 1: Tables A1, A2). At the same time a high number of formulations continue to be marketed without central approval, many of which had not been submitted by manufacturers to the Kokate committee (Additional file 1: Tables A5).

The still inadequate control of FDCs in India flows from the division of responsibility between the centre and the states dating from 1940. In July 2022, as part of a review of obsolete laws and updating existing ones, the government published for consultation a draft of a new Drugs, Medical Devices and Cosmetics Bill “in order to keep pace with changing needs, times, [and] technology” [45]. The proposed bill leaves the division of responsibility in place, and early commentaries suggest it will not meet its objectives [46, 47]. The recommendations that the centre should have sole constitutional responsibility for drug regulation, and should share responsibility for public health [5] remain unaddressed.

Box: Summary of results *First initiative since 2007—*cancellation of manufacturing licences for FDCs issued without prior central approval:

- Limited to a list of 294 FDCs
- Delayed by legal challenge; seven years before the statutory committee reported on its assessments in 2015; plus four more years before banning orders in 2019
- 11 antibiotic FDCs banned in 2019 following assessment by Gupta committee
  - Only 5/11 marketed between 2008–20; their market share by volume, although very small, increased until 2018 and declined after the 2019 ban; eight formulations still on the market in 2020
  - 6/11 not marketed between 2008–2020

*Second initiative since 2013—*assessment of safety and efficacy of FDCs issued manufacturing licences without a prior central approval:

- Limited to formulations submitted by manufacturers for assessment
- Assessment process delayed by industry concerns, then bans in 2016 delayed by legal challenge
- Effective approval: 68 FDC formulations were permitted NOCs in 2015–2020
  - 33.8% (23/68) marketed in 2020, with 10.6% market share of the antibiotic FDC market by volume
  - 41.2% (28/68) not marketed between 2013–2020
- Bans:
  - 36 antibiotic FDCs banned in 2016 and 2017, following assessment by Kokate committee
  - 2016 ban initially stayed, then re-assessment ordered
  - 34 antibiotic FDCs banned and one restricted in 2018 following assessment by Kshirsagar committee
    - 17/34 marketed between 2013–20; their market share started to decline before the 2018 ban when they accounted for 4% of the FDC market; 31 formulations still on the market in 2020
    - 17/34 not marketed between 2013–2020

*Impact on sales of FDCs in 2020*

- 20 FDCs (at least 39 formulations) were marketed despite having been banned in 2018 and 2019
- Although the number of unapproved FDCs and their market share have declined, 60.5% (239/395) of formulations marketed were unapproved, accounting for 15.7% of the FDC sales
- 55.3% of the antibiotic FDC market by volume had a record of prior central approval (compared with 32.0% in 2008), 10.6% were permitted NOCs, and 15.9% were either banned or unapproved

*National List of Essential Medicines (NLEM)—*market share by volume of NLEM-listed combinations increased over study period from 21.2 to 26.7%. 75% (15/20) of NLEM-listed combinations were approved (13 having central approval and two being permitted NOCs).

### Limitations

The analysis was limited to publicly available official documents and by the quality of market sales data. The CDSCO website may not be complete; for example, we did not locate a copy of the Gupta report, only a summary in minutes of a DTAB meeting; and we were unable to find copies of the government’s directions issued to state regulators in November 2007 to cancel licences.

Pharmatrac data reflect sales in private sector which accounted for 69% of the total expenditure on medicines in India in 2017–2018 [48]. Data on public procurement of medicines were not available.

PharmaTrac data misreported and missed some strengths of marketed formulations. As a result, some approved formulations may have been inadvertently coded as unapproved in the sales data and vice versa. We could not determine approval status for almost one-fifth of sales volume in 2020.

### Recommendations

Regulatory enforcement and antimicrobial stewardship should be informed by regular surveillance of the market. Based on such surveillance, there is a clear case, as Sharma acknowledges [5], for considering criminal proceedings against the manufacturers of the 233 formulations marketed in 2020 without a record of prior CDSCO approval in circumstances where such approval was legally required but had not been formally or effectively granted**.** Criminal proceedings should also be considered against the manufacturers of those 39 formulations which were on the market in 2020 but which have been banned. Prescribing only well-evidenced FDCs, such as those listed in NLEM would reduce use of FDCs and facilitate antimicrobial stewardship.

Attention should also be given to the recommendation of the Kokate committee which called for periodic reviews to be undertaken of approved FDCs across all therapeutic areas which have been marketed for more than 10 years (Additional file 1: Tables A8 lists antibiotic FDC formulations). A review of FDCs approved in India which are not recommended by the WHO should also be conducted, and the government should explain why in 2019 it removed the requirement for CDSCO, before giving its approval for new drugs, including FDCs, to be satisfied of their safety and effectiveness. Further research is needed on the control measures and trends in sales of FDCs in other therapeutic areas. More fundamentally, the calls for constitutional reform and modern legislation to ensure transparent coordination between the centre and the states must be heeded.

### Supplementary Information

**Additional file 1: Table A1.** Systemic antibiotic FDC formulations permitted *de facto* approval outside the statutory regime (No Objection Certificate) for continued manufacturing and marketing under 18 month policy decision. **Table A2.** Systemic antibiotic FDCs banned and restricted in use in India, 2016-2019. **Table A3.** Systemic antibiotic FDCs marketed in India, 2008-2020. **Table A4.** FDC formulations banned by Ministry of Health and Family Welfare and still being sold in 2020. **Table A5.** FDC formulations with neither a record of formal approval from CDSCO nor *de facto* approval outside the statutory regime (No Objection Certificate) still being sold in 2020. **Table A6.** NLEM formulations of systemic antibiotic FDCs marketed in 2008-2020, by approval status. **Table A7.** Number, volume and market share of systemic antibiotic FDC formulations marketed in India by NLEM and approval status; 2008-2020. **Table A8.** Systemic antibiotic FDC formulations marketed in India for 10 and more years.

## Data Availability

The data that support the findings of this study are available from PharmaTrac but restrictions apply to the availability of these data, which were used under license for the current study, and so are not publicly available (https://www.aiocdawacs.com/(S(0vvl5kmxue1kguc4ljbksdcc))/ProductDetail.aspx). In the publicly available study appendices, we provide full details of the systemic antibiotic formulations and aggregate volume data collated from PharmaTrac.

## Abbreviations

CDSCO: Central Drugs Standard Control Organisation
DCG(I): Drugs Controller General (India)
DTAB: Drugs Technical Advisory Board
FDC: Fixed-dose combination
MoHFW: Ministry of Health and Family Welfare
NLEM: National List of Essential Medicines
NOC: No Objection Certificates
PMO: Prime Minister’s Office
SD: Single drug
SU: Standard unit
WHO: World Health Organization
